# The effect of preoperative urine culture and bacterial species on infection after percutaneous nephrolithotomy for patients with upper urinary tract stones

**DOI:** 10.1038/s41598-022-08913-7

**Published:** 2022-03-22

**Authors:** Zesong Yang, Ding Lin, Yun Hong, Minxiong Hu, Wanghai Cai, Honghong Pan, Qiuyan Li, Jiexiang Lin, Liefu Ye

**Affiliations:** Department of Urology, Fujian Provincial Hospital, Shengli Clinical Medical College of Fujian Medical University, Fuzhou, 350001 China

**Keywords:** Diseases, Risk factors, Signs and symptoms, Urology

## Abstract

To study the relationship between preoperative urine culture, bacterial species and infection after percutaneous nephrolithotomy in patients with upper urinary tract stones, and summarize the clinical characteristics of different bacterial infections. From January 2014 and January 2020, 963 patients with upper urinary tract stones who underwent PCNL in the department of urology of Fujian provincial hospital were included in the study. Information included the patient’s age, gender, weight, diabetes, chronic disease history, urine routine, preoperative urine culture results, stone size, number of stones, hydronephrosis level, operation time, body temperature, heart rate, blood pressure, breathing rate, hemoglobin, serum creatinine, bilirubin, platelets and whether there was preoperative infection were recorded. 141 patients (14.6%) had a positive urine culture before surgery, and 7 of them had multiple bacterial infections. The most common pathogenic bacteria was Escherichia coli, followed by Enterococcus and Klebsiella pneumoniae. A total of 74 cases (7.7%) of 963 patients with infection after PCNL occurred, 24 cases (32.4%) of infected patients progressed to urinary septic shock. Univariate analysis shown that the probability of infection in patients with long operation time and positive urine culture was significantly higher, and the difference was statistically significant. Further multivariate logistic regression analysis shown that positive urine culture before operation and long operation time were independent risk factors for infection after PCNL. Among the 29 patients with septic shock, 18 cases (62.1%) had a positive urine culture before surgery. The incidence (43.9%) of postoperative infection in Escherichia coli positive patients was significantly higher than that in the negative group, and the difference was statistically significant. The rate of patients with Escherichia coli infection progressing to septic shock was 9 cases (60%). 2 patients with Enterococcus faecium infection and 2 patients with Klebsiella pneumoniae infection all progressed to septic shock. The age of patients with post-PCNL infection caused by Escherichia Coli, Enterococcus faecium and Klebsiella pneumoniae were 58.53 ± 11.73 years, 76.5 years and 74 years.The body temperature of patients with post-PCNL infection caused by Escherichia Coli, Enterococcus faecium and Klebsiella pneumoniae were 39.10 ± 0.25 °C, 39.45 °C and 38.65 °C. The highest pct value of patients with post-PCNL infection caused by Escherichia Coli, Enterococcus faecium and Klebsiella pneumoniae were 80.62 ± 31.45 ng/mL, 24.32 ng/mL and 8.45 ng/mL. The nitrite positive rate of patients with post-PCNL infection caused by Escherichia Coli, Enterococcus faecium and Klebsiella pneumoniae were 64.51%, 16.6% and 0. Postoperative infection of PCNL is significantly correlated with positive preoperative urine culture, and positive preoperative urine culture is an independent risk factor for postoperative infection. The most common pathogen of postoperative infection of PCNL is Escherichia coli, followed by Enterococcus and Klebsiella pneumoniae. Patients with Escherichia coli infection are often positive for nitrite before surgery, mainly manifested by high fever, and PCT is significantly increased (often exceeded 100 ng/ml). Enterococcus faecium and Klebsiella pneumoniae infections mostly occur in elderly patients and often progress to septic shock. Patients with Enterococcus faecium infection have a high fever, and the PCT value is significantly higher (often exceeded 20 ng/ml). Patients with Klebsiella pneumoniae infection have a moderate fever, and the PCT value generally does not exceeded 10 ng/ml. Long operation time is another independent risk factor for PCNL infection.

## Introduction

Urinary calculi is one of the common diseases of urology, which includes upper urinary tract stones and lower urinary tract stones. Among them, upper urinary tract stones account for the majority of cases. The morbidity and prevalence rates were increasing all over the world^1^. Epidemiological surveys in Europe and the United States shown that the prevalence of urinary stones can be as high as 7.1–10.6%^2^. The prevalence rate of urinary stones in China was as high as 6.5%. In southern coastal provinces, the prevalence rate was significantly higher than other provinces due to the high proportion of calcium and magnesium ions in people's daily drinking water^3^.

Percutaneous nephrolithotomy (PCNL) is one of the main treatments for upper urinary tract stones, which has the advantages of less trauma and high efficiency. Although PCNL can effectively remove stones, perioperative complications still occur from time to time. Postoperative complications of PCNL include bleeding, infection, surrounding tissue damage, thromboembolism, etc^4^. As a common complication, postoperative infection can cause septic shock if it is not treated in time. Urinary septic shock progresses quickly and is difficult to deal with, and the mortality rate can reach 25–60%^5^. At present, there is no unified guideline for the risk factors related to postoperative infection. Research reported indicate that the main factors that cause postoperative infection include preoperative urine culture positive, stone bacterial culture positive, stone burden, female, elderly, diabetes, urinary tract obstruction, etc^5–7^.

The positive urine culture before operation is closely related to the occurrence of postoperative infection. Studies have shown that even if antibiotics are used before surgery to transform the urine with bacteria into a sterile state, infection after PCNL cannot be avoided, and the infection is obviously related to preoperative bacteria in the urinary tract^8^. The establishment of bacterial spectrum is of great significance in preventing postoperative infections^9^. At present, there is no clear conclusion about the relationship between the bacterial species in the urine before surgery and the infection after PCNL. Therefore, the purpose of this study is to explore the correlation between different bacterial species and postoperative infection of PCNL, and to further summarize the postoperative infection characteristics of various kinds of bacteria, so as to provide a reference for clinical treatment.

## Materials and methods

### Patients

From January 2014 and January 2020, 963 patients with upper urinary tract stones who underwent PCNL in the department of urology of Fujian provincial hospital were included in the study. Computed tomography urography (CTU) images of the patients were acquired for all the study patients. Patients with tumors, blood system diseases, immune system diseases, patients with other system infections, patients undergoing second-stage PCNL, patients with severe cardiovascular and cerebrovascular diseases, and patients undergoing other operations during the same period were excluded. Patients with positive urine bacteria culture had been treated with sensitive antibiotics until the urine bacteria culture turned negative, and patients with diabetes had been treated with insulin until the fasting plasma glucose had been controlled below 8 mmol/L.

### Data collection

All patients were confirmed to have unilateral renal stones or upper ureteral stones before operation. The following data were recorded: (1) General information: the patient’s age, gender, weight, diabetes, and chronic disease history. (2) Laboratory examination: urine routine, preoperative urine culture results. (3) Imaging examination: stone size, number of stones, hydronephrosis level. (4) Surgery information: operation time. (5) Postoperative information: body temperature, heart rate, blood pressure, breathing rate; hemoglobin, serum creatinine, bilirubin, platelets, whether there was infection. Procalcitonin (PCT) recorded the highest postoperative value of the patient.

#### Criteria for infection

One of the following manifestations after surgery was considered to be postoperative infection of PCNL: (1) positive blood culture. (2) Positive urine culture. (3) Body temperature above 38.5 °C, and excluded other systemic infections and fever caused by other factors.

The infection was evaluated using the third-generation diagnostic criteria for sepsis developed by the European society of intensive care medicine in 2016^10^: that is, when infection exists, the sequential organ failure score ≥ 2 points indicates the presence of sepsis. On this basis, vasopressors need to be used to maintain mean arterial pressure ≥ 65 mmHg or serum lactate level > 2 mmol/L when blood volume is reduced, which is considered as septic shock.

#### Hydronephrosis classification

Ipsilateral hydronephrosis was graded 0–4 according to CTU images: grade 0- no caliceal or pelvic dilatation, grade 1—pelvic dilatation only, grade 2—mild caliceal dilatation, grade 3—severe caliceal dilatation and grade 4—caliceal dilatation accompanied by renal parenchymal atrophy. Grade 3 and grade 4 hydronephrosis were defined as high-grade hydronephrosis^11–13^.

#### Classification of fever

According to the level of fever, it can be divided into (1) low fever (37.3–38 °C). (2) Moderate fever (38.1–39 °C). (3) High fever (39.1–41 °C). (4) Super high fever (above 41 °C).

### Surgery

After the patients were placed in the lithotomy position. A 5F ureteral catheter was inserted into the ureteron the operation side and accessed ureteropelvic junction over a Cook 0.38 guidewire using cystoscopy. Then, the patients were turned into a prone position. Eighteen-gauge Chiba needles were used to achieve target renal calyx under ultrasound guidance. A Cliny 0.38 J type guidewire was placed into the renal pelvis on the side of surgery. Fascial dilators were used for dilation over the guidewire, and an F24 Amplatz sheath was placed in the kidney. Renal access was gained through the Amplatz sheath using a nephroscope (Wolf 8964.401 8F) device. A pneumatic and ultrasonic endoscopic lithotripter (Electro Medical Systems/EMS-IV) was used to dust and remove upper ureteral stones. An F20 nephrostomy tube was placed in each of the 963 patients.

### Statistical analysis

SPSS 25.0 software was used for statistical analysis. Measurement data were expressed by mean ± standard deviation or median and interquartile range, enumeration data were expressed by number of cases and percentages. Independent sample t test was used for comparison of normal distribution measurement data, and rank-sum test was used for non-normal distribution measurement data. The comparison of enumeration data adopted the chi-square test. The two-category unconditional multivariate Logistic regression model was used to study the relationship between the two factors. A P value of < 0.05 was considered statistically significant.

### Research involving human participants

Informed consent was obtained from all subjects. The study was approved from by the Ethics Committee of Fujian Provincial Hospital and People's Hospital of Zhejiang Province. And we certify that the study was performed in accordance with the ethical standards as laid down in the 1964 Declaration of Helsinki and its later amendments or comparable ethical standards.

## Results

All patients underwent PCNL by experienced physicians. General information about the patients included in the study and univariate analysis results of preoperative risk factors were summarized in Table 1.

**Table 1:** General information and univariate analysis results of preoperative risk factors about the patients.

Parameters	Infection (n = 74)	Non-infection (n = 889)	t/χ^2^ value	P value
**Gender**				
Male, n	39	559	3.006	0.08
Female, n	35	330		3
Weight, kg	62.68 ± 10.64	63.00 (55.00, 70.00)	–	0.729*
Age, years	56.86 ± 12.99	55.00 (47.00, 63.00)	–	0.095*
**Diabetes**				
Yes, n	5	109	1.983	0.159
No, n	69	780		
**Chronic disease**				
Yes, n	27	250	2.333	0.127
No, n	47	639		
**High-grade hydronephrosis**				
Yes, n	27	405	2.272	0.132
No, n	47	484		
Size of stones, mm	21.64 ± 9.89	19.00 (14.00, 25.00)	–	0.261*
Operating time , minutes	114.00 (80.00, 151.25)	100.00 (75.00, 130.00)	–	0.024*
**Preoperative urine culture**				
Positive, n	29	112	38.648	< 0.001
Negative, n	45	777		

*The result was obtained using the rank-sum test.

In this study, a total of 74 cases (7.7%) of 963 patients with infection after PCNL occurred, 24 cases (32.4%) of infected patients progressed to urinary septic shock, but no patient died. Univariate analysis shown that the probability of infection in patients with long operation time and positive urine culture was significantly higher, and the difference was statistically significant. Further multivariate logistic regression analysis shown that positive urine culture before operation and long operation time were independent risk factors for infection after PCNL (Table 2).

**Table 2:** Multivariate analysis results of risk factors for infection after PCNL.

Parameters	β	SE	Wald	OR	95% CI	P value
Preoperative Urine culture	1.431	0.261	29.964	4.184	2.506–6.985	< 0.001
Operating time	0.006	0.003	4.061	1.006	1.000–1.011	0.044

As it was shown in Table 3, among the 29 patients with septic shock, 18 cases (62.1%) had a positive urine culture before surgery, shown that patients with a positive urine culture before surgery were more likely to progressed to septic shock than those with a negative urine culture, and the difference was statistically significant.

**Table 3:** Univariate analysis results of preoperative risk factors about the patients with postoperative infection progressed to septic shock.

Parameters	Septic shock (n = 24)	Non-shock (n = 50)	t/χ^2^ value	P value
**Gender**				
Male, n	10	29	1.736	0.188
Female, n	14	21		
Weight, kg	61.61 ± 9.46	63.18 ± 10.91	0.606	0.546
Age, years	57.13 ± 15.03	56.74 ± 12.05	− 0.119	0.906
**Diabetes**				
Yes, n	2	3		0.657^Δ^
No, n	22	47		
**Chronic disease**				
Yes, n	11	16	1.339	0.247
No, n	13	34		
**High-grade hydronephrosis**				
Yes, n	11	16	1.339	0.247
No, n	13	34		
Size of stones, mm	23.00 (18.00, 29.75)	19.00 (10.00, 30.00)	–	0.069*
Operating time , minutes	120.00 (88.75, 161.25)	100.00 (80.00, 151.25)	–	0.236*
**Preoperative urine culture**				
Positive, n	18	11	19.114	< 0.001
Negative, n	6	39		

^Δ^The result was obtained using Fisher's exact probability test.

*The result was obtained using the rank-sum test.

Among the 963 patients, 141 (14.6%) had a positive urine culture before surgery, and 7 of them had multiple bacterial infections. Bacteria species and related statistical analysis results were shown in Table 4. The most common pathogenic bacteria was Escherichia coli, followed by Enterococcus and Klebsiella pneumoniae. The incidence (43.9%) of postoperative infection in Escherichia coli positive patients was significantly higher than that in the negative group, and the difference was statistically significant. The rate of patients with Escherichia coli infection progressing to septic shock was 9 cases (60%). 2 patients with Enterococcus faecium infection and 2 patients with Klebsiella pneumoniae infection all progressed to septic shock. The clinical characteristics of post-PCNL infection caused by them were shown in Table 5.

**Table 4:** Species of urine cultured bacteria and related statistical analysis results.

Bacteria	Case, n (%)	Postoperative infection, n	χ^2^ value	P value	Septic shock, n
Escherichia Coli	62 (43.9%)	15	23.034	< 0.001^Δ^	9
Enterococcus faecalis	13 (9.2%)	1	–	–	0
Klebsiella pneumoniae	8 (5.6%)	2	1.392	0.238	2
Proteus mirabilis	7 (4.9%)	1	–	–	1
Enterobacter cloacae	7 (4.9%)	1	–	–	0
Enterococcus faecium	6 (4.2%)	2	2.552	0.110	2
Streptococcus agalactiae	5 (3.5%)	1	–	–	0
Streptococcus anginosus	3 (2.1%)	1	–	–	0
Pseudomonas aeruginosa	3 (2.1%)	0	–	–	0
Streptococcus mitis	3 (2.1%)	0	–	–	0
Acinetobacter baumannii	2 (1.4%)	0	–	–	0
Serratia marcescens	2 (1.4%)	0	–	–	0
Aeromonas hydrophila	2 (1.4%)	0	–	–	0
Staphylococcus hominis subsp	2 (1.4%)	1	–	–	0
Staphylococcus epidermidis	2 (1.4%)	0	–	–	0
Streptococcus pastoris	1 (0.7%)	0	–	–	0
Streptococcus mutans	1 (0.7%)	0	–	–	0
Klebsiella odorifera	1 (0.7%)	1	–	–	1
Staphylococcus saprophyticus	1 (0.7%)	0	–	–	0
Staphylococcus aureus	1 (0.7%)	1	–	–	1
Pantoea agglomerans	1 (0.7%)	0	–	–	0
Haemophilus influenzae	1 (0.7%)	0	–	–	0
Multiple bacterial	7 (4.9%)	2	–	0.171	2

^Δ^The result was obtained using Fisher's exact probability test.

**Table 5:** The clinical characteristics of post-PCNL infection caused by different bacteria.

Parameters	Escherichia coli	Enterococcus faecium	Klebsiella pneumoniae
Age, years	58.53 ± 11.73	76.5	74
**Gender**			
Male, n	2	1	2
Female, n	13	1	0
Rate of diabetes, %	26.67	50	50
Rate of infection, %	24.19	25.00	33.33
Rate of infection progressed to septic shock, %	60	100	100
body temperature, °C	39.10 ± 0.25	39.45	38.65
Highest pct value, ng/mL	80.62 ± 31.45	24.32	8.45
Nitrite positive rate, %	64.51	16.6	0

## Discussion

Upper urinary tract stones can cause urinary tract obstruction and provide a favorable environment for the growth of pathogenic bacteria, and the stones are a favorable place for bacterial colonization. At the same time, bacteria can further promote the growth of stones. In PCNL surgery, it is necessary to pressurize and infuse fluid into the renal pelvis to maintain a clear vision. Excessive internal pressure in the renal pelvis causes bacteria and endotoxins to flow back into the blood circulation, triggering an inflammatory response^14^.

The presence of bacteria in the urinary tract before surgery is an important cause of infection after PCNL. Even if antibiotics are used before surgery, the bacteria hidden in the stones are difficult to remove. Previous studies have reported that the incidence of postoperative infection in patients with positive urine culture before surgery was as high as 18.2% among patients undergoing PCNL^15^. Erdil et al.^16^conducted a retrospective analysis of 317 patients in a study and shown that in the postoperative infection group, 33.9% of the patients with positive urine culture before surgery, while the uninfected group was accounted for only 9.8%, the difference was statistically significant. Lee et al.^17^ conducted a sepsis risk factor study on 2196 patients undergoing PCNL, and the results shown that a positive urine culture before surgery was an independent risk factor for postoperative septic shock.

In this study, 29 of the 141 preoperative urine culture-positive patients had postoperative infections, and the infection incidence was 20.57%. The incidence of these infected patients progressing to septic shock was 62.07%, which was significantly higher than for patients with negative urine culture before operation, the chi-square test shown a P value < 0.001, and the difference was statistically significant. The multi-factor analysis of postoperative infection risk factors shown that preoperative urine culture (OR = 4.184) was an independent risk factor for postoperative infection, which is similar to previous research results^15–17^. Therefore, patients with positive urine culture have a higher risk of infection after PCNL and are likely to progress to septic shock. Taking preoperative urine culture as a necessary preoperative examination and timely anti-infective treatment can help reduce the occurrence of postoperative infection.

It should be noted that preoperative urine culture takes a long time, and it often takes 2–3 days to obtain the culture results. And urine culture also has the disadvantages of low sensitivity and possible underreporting. According to research reported, the positive rate of urine culture was low, depending on the population and environment, it may be only 20%^18^. Therefore, it is necessary to know the common urinary tract bacteria and their corresponding infection characteristics, which will help clinical diagnosis and treatment.

In this study, the most common preoperative urine culture was Escherichia coli, which was consistent with previous studies^19,20^. Enterococcus and Klebsiella pneumoniae came in second place. Positive Escherichia coli is significantly associated with post-PCNL infection. Patients with positive Enterococcus faecium and Klebsiella pneumoniae were more likely to had postoperative infections than patients with positive Escherichia coli (25%, 33.3% vs 24.19%). However, Enterococcus faecium and Klebsiella pneumoniae have not obtained statistical analysis results related to infection after PCNL, this may be related to the small number of samples.

Escherichia coli is the main pathogen of urinary tract infections^21^, more than 90% of urinary tract infections are caused by Escherichia coli^22^. In this study, the incidence of postoperative infection in patients with preoperative urine culture of Escherichia coli positive was as high as 24.1%, and the rate of progression to septic shock was as high as 60%. High fever, high PCT value and positive urine nitrite were the main characteristics of Escherichia coli infection. The maximum value of PCT in patients infected with Escherichia coli often exceeded 100 ng/ml, and the average value of the peak reached 80.62 ng/ml.

Enterococcus was another common pathogen of urinary tract infections. Studies have reported that Enterococcus has surpassed Escherichia coli, becoming the first pathogenic bacteria for urinary tract infections^23^. In urinary tract infections, the pathogens of Enterococcus were mainly Enterococcus faecalis and Enterococcus faecium. Compared with Enterococcus faecium, Enterococcus faecalis had lower virulence. In this study, only 1 out of 13 patients with Enterococcus faecalis-positive had an infection, and no sepsis occurred. However, 2 out of 6 patients with Enterococcus faecium positive developed postoperative infection, and all of them progressed to sepsis. High fever can also occur in patients with Enterococcus faecium infection. Although the PCT value is also increased, its peak value often exceeds 20 ng/ml, but significantly lower than that of patients with Escherichia coli infection. What needs to be vigilant was that patients with Enterococcus faecium infection were prone to septic shock, which may be related to it being a conditional pathogen and often infecting elderly patients with poor immunity.

Klebsiella pneumoniae was another common pathogenic bacteria, so it was also more common in the elderly and easily induces septic shock. The difference was that Klebsiella pneumoniae infection was characterized by moderate fever, and the peak value of PCT was lower than that of patients with Escherichia coli and Enterococcus faecium infection. In this study, the peak PCT of patients with Klebsiella pneumoniae infection did not exceeded 10 ng/ml. Lin et al.^24^ found that the adhesion factor of Klebsiella pneumoniae had strong invasiveness and hiding ability, which can hid in the bladder urothelial cells, so that Klebsiella pneumoniae had the ability to immune escape and resisted urine erosion. At the same time, due to the increase of its drug resistance, it leaded to repeated infections, especially in patients with poor plasma glucose control in diabetes.

The different clinical characteristics of infections caused by Escherichia Coli, Enterococcus faecium and Klebsiella pneumoniae may be related to their mechanisms, but no studies have revealed the difference at present.

The analysis of this study shown that the incidence of postoperative infections was higher in patients with longer operation time. Among patients with septic shock, the proportion of women and patients with longer operation time was larger. Long operation time was an independent risk factor for infection after PCNL, and its OR value was 1.006.

There were various limitations of this study. Since our study was a retrospective design, the data may be biased. Te number of patients included in the study was limited due to the inclusion of a single center. Further prospective studies on larger patient series were needed. In addition, Intraopetric pressure is a key-point for post-operative infections for PCNL. However, this was a retrospective study, so data on IPP were not available.

## Conclusion

Postoperative infection of PCNL is significantly correlated with positive preoperative urine culture, and positive preoperative urine culture is an independent risk factor for postoperative infection. The most common pathogen of postoperative infection of PCNL is Escherichia coli, followed by Enterococcus and Klebsiella pneumoniae. Patients with Escherichia coli infection are often positive for nitrite before surgery, mainly manifested by high fever, and PCT is significantly increased (often exceeding 100 ng/ml). Enterococcus faecium and Klebsiella pneumoniae infections mostly occur in elderly patients and often progress to septic shock. Patients with Enterococcus faecium infection have a high fever, and the PCT value is significantly higher (often exceeding 20 ng/ml). Patients with Klebsiella pneumoniae infection have a moderate fever, and the PCT value generally does not exceed 10 ng/ml. Long operation time is another independent risk factor for PCNL infection.

## Supplementary Information

Supplementary Information.

## Data Availability

All data generated or analysed during this study are included in this published article (and its supplementary information files S1).
